# Indenting soft samples (hydrogels and cells) with cantilevers possessing various shapes of probing tip

**DOI:** 10.1007/s00249-020-01456-7

**Published:** 2020-08-17

**Authors:** Joanna Zemła, Justyna Bobrowska, Andrzej Kubiak, Tomasz Zieliński, Joanna Pabijan, Katarzyna Pogoda, Piotr Bobrowski, Małgorzata Lekka

**Affiliations:** 1 Institute of Nuclear Physics Polish Academy of Sciences, PL-31342 Kraków, Poland; 2 Institute of Metallurgy and Materials Science Polish Academy of Sciences, PL-30059 Kraków, Poland

**Keywords:** Atomic force microscopy (AFM), Cancer cell mechanics, Indenter geometry

## Abstract

The identification of cancer-related changes in cells and tissues based on the measurements of elastic properties using atomic force microscopy (AFM) seems to be approaching clinical application. Several limiting aspects have already been discussed; however, still, no data have shown how specific AFM probe geometries are related to the biomechanical evaluation of cancer cells. Here, we analyze and compare the nanomechanical results of mechanically homogenous polyacrylamide gels and heterogeneous bladder cancer cells measured using AFM probes of various tip geometry, including symmetric and non-symmetric pyramids and a sphere. Our observations show large modulus variability aligned with both types of AFM probes used and with the internal structure of the cells. Altogether, these results demonstrate that it is possible to differentiate between compliant and rigid samples of kPa elasticity; however, simultaneously, they highlight the strong need for standardized protocols for AFM-based elasticity measurements if applied in clinical practice including the use of a single type of AFM cantilever.

## Introduction

The biological functionality of cells and tissues is strongly correlated with their nanomechanical properties, usually quantified by Young’s modulus—a physical parameter describing the material’s resistance to elastic deformations (Sneddon 1965). One of the tools for studying cell mechanics in conditions close to physiological ones is atomic force microscopy (AFM). In most cases, for the data recorded using AFM-based force spectroscopy, Young’s modulus is evaluated in the frame of Hertz contact mechanics, which describes the deformation (indentation) of two purely elastic spheres. The model can be extended into a case when a stiff sphere indents an infinitely thick, isotropic and elastic half-space in the absence of adhesion within the contact area (Hertz 1881). Sneddon’s modifications resolved the problem of indenter geometry by solving the Boussinesq problem, i.e., finding the elastic state in a linearly elastic isotropic half-space, subjected to a concentrated load applied in a point of its boundary plane and perpendicular to it, assuming various axisymmetric geometries of the indenters of various shapes (Sneddon 1965). One of the main requirements in AFM is that sample height should be large enough to provide conditions avoiding the influence of the underlying stiff supports. This implies that the indentation depth should be below c.a. 10% of the sample height. Furthermore, a typical shape of the probing tip is a four-sided pyramid that is approximated either by a cone or paraboloid (Schillers et al. 2017). Although more relevant, less frequently, a Hertz contact model with the extension for the four-sided pyramidal indenter geometry is used (Weber et al. 2019). The use of spherical indenters to data collected with a spherical AFM probe does not introduce a large discrepancy (Lin and Horkay 2008; Kim et al. 2013; Guz et al. 2014; Puricelli et al. 2016). Instead discrepancies arise in the case of data recorded with pyramidal probes (Radmacher 2007; Sirghi et al. 2008; Wang et al. 2015; Kilpatrick et al. 2015; Rianna and Radmacher 2016).

A single cell cannot be treated as a purely elastic material as various studies show its viscoelastic nature (e.g., Nawaz et al. 2012). However, despite the ongoing development in the modeling of the mechanical properties of single cells, the Hertz contact theory is still dominant and widely employed in AFM-based data analysis due to its simplicity. As a consequence, measurements of relative Young’s modulus are used to quantify changes in biological samples (relativeness of the elastic modulus for biological samples has been discussed in Lekka et al. 2012; Lekka 2016). In the majority of pathological conditions, cells alter mechanical properties as has been reported for cancer (Lekka et al. 1999, 2012; Faria et al. 2008; Prabhune et al. 2012; Ketene et al. 2012; Kim et al. 2013; Chiou et al. 2013; Ramos et al. 2014; Zhao et al. 2015; Lekka 2016; Rianna and Radmacher 2016; Alibert et al. 2017). So far, only a few papers have reported on the effect of probe shape on the determined Young’s modulus (Rico et al. 2005; Chiou et al. 2013; Kim et al. 2013; Lekka 2016; Managuli and Roy 2017; Alcaraz et al. 2017; Sokolov and Dokukin 2017). Reported results indicate that cells probed with cantilevers possessing pyramidal tips mounted at the free end reveal larger elastic moduli (cells seem to be more rigid) as compared to measurements carried out on the same cell type with spherical probes (Rico et al. 2005; Carl and Schillers 2008; Managuli and Roy 2017; Alcaraz et al. 2017; Sokolov and Dokukin 2017; Jorba et al. 2017; Giménez et al. 2017). In most cases, a comparison was carried out for one cell type such as Chinese hamster ovary cells (Carl and Schillers 2008) or fibroblasts (Chiou et al. 2013); thus, it still is not clear how probe geometry affects the nanomechanics-based identification of cancer cells. This is important for improving the diagnostic significance of AFM-based elasticity measurements, especially given that the absolute value of Young’s modulus is difficult to obtain. Our earlier measurements have revealed that human bladder cancer cells are significantly more deformable as compared to non-malignant bladder cancer cells (Lekka et al. 1999, 2019; Ramos et al. 2014). Therefore, we have chosen two cell lines, namely, HCV29 (non-malignant cell cancer of ureter), and HT1376 (bladder carcinoma) for this study. Importantly, these cells are characterized by a distinct organization of actin filaments, as the main cytoskeletal component responsible for the elastic properties of the cells (Ketene et al. 2012; Ramos et al. 2014). Non-malignant cells have a well-organized actin cytoskeleton with developed thick, long fibers accompanied by short thin filaments. Carcinoma HT1376 cells display only short actin filaments (Lekka et al. 2019). Observed differences in actin cytoskeleton make these cells suitable as a cellular model to quantify differences in cellular deformability using indenters of various geometries.

In our studies, we compare nanomechanical properties of soft samples (polyacrylamide hydrogels and cells) based on measurements carried out with AFM probes of various tip geometry, including symmetric and non-symmetric pyramids and a sphere. Our findings show the tip shape-dependent variability of Young’s modulus; however, while working within the range of moduli characteristic for living cells, it is still possible to differentiate between compliant and rigid samples. Simultaneously, these results highlight the need for standardized protocols in choosing cantilever type if applied in clinical practice.

## Materials and methods

### Coverslips for hydrogels deposition and polymerization

Bottom coverslips (Ø15 mm, Thermo Scientific) were sonicated in acetone, subsequently rinsed with dH_2_O, and dried. Afterward, they were placed in a vacuum desiccator for 1 h and 3-aminopropyltriethoxysilane (APTES, Sigma-Aldrich) vapor deposition occurred. Then, the slides were immersed in 0.5% glutaraldehyde (GA, Sigma-Aldrich) in deionized water for an hour and dried. Top coverslips (Ø22 mm, Thermo Scientific) were coated with 5% SurfaSil Solution (Sigma-Aldrich) in acetone for 10 s and subsequently rinsed in acetone and methanol and dried.

### Polyacrylamide gels

Stock solutions of 40% acrylamide (Sigma-Aldrich) and 2% bis-acrylamide (Sigma-Aldrich) prepared in dH2O were used to make polyacrylamide gel (PA) precursor solutions. Polyacrylamide water solutions (1 ml total volume) with final acrylamide concentration of 5% and 7% were prepared by mixing 125 μl and 175 μl of acrylamide precursor solution with 200 μl of bis-acrylamide (bis-A) precursor solution (0.4%), respectively. Before crosslinking, initiator (10 μl of 10% ammonium persulfate water solution) and accelerator (1.5 μl of tetramethylethylenediamine (TEMED, Fisher Scientific) were added to the polyacrylamide solutions and degassed in a vacuum desiccator. Then, after gentle mixing with the pipette, 60 μl drop of the final solution was placed on a bottom coverslip and covered with a SurfaSil modified coverslip, which was removed after 20 min polymerization time. Polyacrylamide gels were placed in dH_2_O and stored at a temperature of 4 °C prior to examination. Two samples per each case were prepared in one batch for the AFM measurements (in total 30–40 elasticity maps were recorded per each case). This approach resulted from the fact that there might be discrepancies in the mechanical properties of PA gels among different batches, although the same preparation protocol is used (Denisin and Pruitt 2016). The reason for choosing these acrylamide concentrations was to obtain gel samples characterized by a similar range of Young’s modulus as cells are. The thickness of PA gel samples was 1–2 mm.

### Cell cultures

Two human cell lines were chosen for the study, i.e., non-malignant cell cancer of ureter (HCV29, Institute of Experimental Therapy, Wrocław, Poland) and urinary bladder carcinoma cell line (HT1376, grade III, ATCC, LGC Standards). HCV29 cells were cultured in RPMI-1640 (Sigma) supplemented with 10% fetal bovine serum (FBS, Sigma) and HT1376 cell line was cultured in Eagle’s medium (EMEM, LGC Standards) supplemented with 10% FBS (LGC Standards). The cells were grown on glass coverslips placed inside the polystyrene Petri dish at 37 °C in 95% air/5% CO_2_ atmosphere. The relative humidity was kept above 98%. For elasticity maps, HCV29 cells were grown in the tissue culture dish (Ø34 mm, TPP^®^). The AFM measurements were carried out after 48 h culture, in the corresponding media, at room temperature. With each cantilever type, ~ 30 individual cells were measured. The height of the cells within the nuclear region was about 7–10 μ.

### AFM-based force spectroscopy

Force spectroscopy measurements were conducted using XE120 AFM (Park Systems, South Korea) equipped with a liquid cell setup. Various silicon nitride cantilevers with mounted probing tips of diverse geometries were employed. They can be divided into three groups, namely, (i) symmetric pyramidal probes (MSCT-AUH, Veeco; customized PNP, Nanosensors; OTR4, Bruker); (ii) non-symmetric pyramidal probes (MSNL&MLCT, Bruker); and (iii) a pyrex-nitride colloidal probes (sQube, CP-PNP-SiO-C-5, NanoAndMore). Nominal values of half open-angles, cantilever spring constants, and radii of curvature are included in Table 1. Spring constants of the cantilevers were calibrated using the thermal noise method in the air (Schillers et al. 2017). Photodetector sensitivity, i.e., the inverse of the slope, was obtained by fitting a line to the slope of the force curve acquired on the stiff substrate (glass or Petri dish). Measurements were conducted on PA gels and cells. Both cells and PA gels were measured with the same cantilevers. Force curves were recorded within a scan area of 6 μm × 6 μm, within which a grid of 8 × 8 points was set. In total, 64 force curves were acquired (in the case of cells, a nuclear region was probed). The force–distance curves were recorded at an approach velocity of 8 μm/s, the maximum force of 7 nN, and a force curve length of 4 μm.

**Table 1:** Characteristic parameters of one set of AFM probes used in the experiment

Cantilever	*h* [μm]	*h*_nom_ [μm]	*α*_nom_ [°] (R[nm])	*α*_SEM_ [°]	*ν*_nom_ [kHz]	*ν*_meas_ [kHz]*	*k*_nom_ [N/m]	*k*_meas_ [N/m]*
MLCT	4.49	2.5–8.0	23 (20)	23	7	8.28 ± 0.03**	0.01	0.014 ± 0.001***
MSNL	4.40	2.5–8.0	22.5 (2)	23	7	8.27 ± 0.02	0.01	0.014 ± 0.001
PNP customized	3.31	3.5	36 (40)	37	13	16.09 ± 0.21	0.03	0.054 ± 0.014
OTR 4	3.09	2.5–3.5	36 (15)	37	11	12.23 ± 0.05	0.02	0.025 ± 0.002
MSCT	3.13	2.5–8.0	36 (20)	37	8	8.27 ± 0.02	0.01	0.014 ± 0.001

Cantilever	*h* [μm]	*h*_nom_ [μm]	*Ø*_nom_ [µm]	*Ø*_SEM_ [μm]	*ν*_nom_ [kHz]	*ν*_meas_ [kHz]*	*k*_nom_ [N/m]	*k*_meas_ [N/m]*
sQube	–	–	6.62	6.4	17	16.22 ± 0.36	0.08	0.046 ± 0.002

Opening angle (i.e., back angle, *α*), height (*h*), the radius of curvature (*R*), and sphere diameter (*Ø*) were estimated based on SEM images with an accuracy of 1%

*n = 3; **error is the half-width taken at half height; ***standard deviation

### Young’s modulus determination

**Fig. 1:**
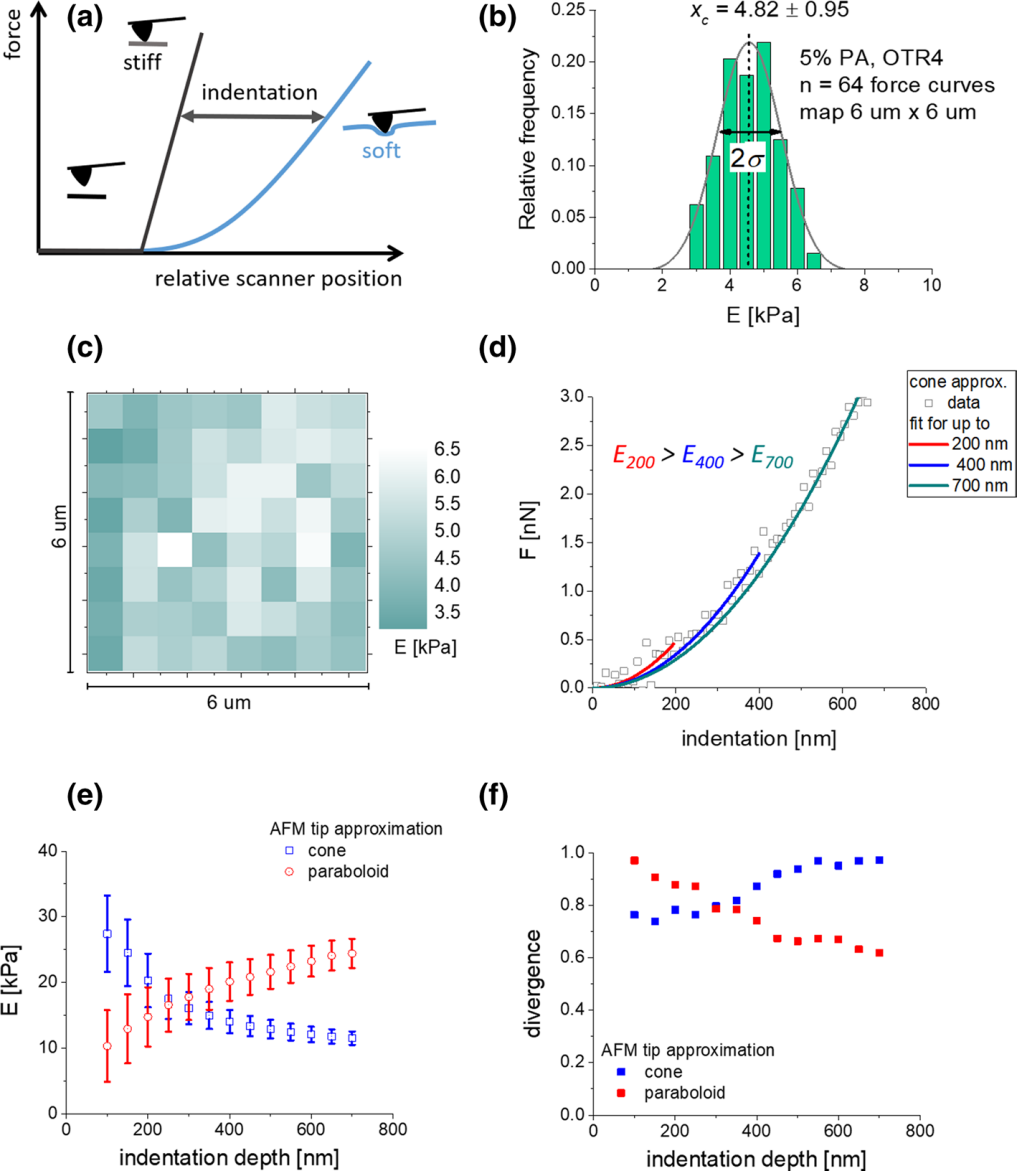
**a** Schematic illustration of AFM-based elasticity measurements carried out for soft samples. Young’s modulus is derived from force-versus-indentation curves being the subtraction results of reference (stiff; glass coverslip surface) and sample (soft; cells or polyacrylamide hydrogels) force curves. **b** Exemplary histogram showing Young’s modulus distribution obtained for (5% PA/0.4% bis-A) hydrogel sample probes over a squared scan area of 6 µm × 6 µm (*n* = 64 force curves; sampling interval Δ*E* = 0.5 kPa); measured with OTR4 probe. The final modulus value was obtained from a Gaussian fit (*E* = 4.82 ± 0.95 kPa). **c** The corresponding 2D elasticity map (force volume). **d** Indentation depth-dependent fitting of the Hertz model to raw data for 5% PA gels. **e** Young’s modulus dependence on indentation depth obtained by fitting a theoretical model assuming either cone or paraboloid shape of the AFM tip. Each point represents the fitted value of the modulus and standard error (from the fit). **f** Divergence calculated for the same data as in **e**

Individual force curve is a relation between a cantilever deflection and a relative scanner position. The deflection of the cantilever is converted into a force by multiplying it by cantilever spring constant. An indentation depth is obtained by subtracting a calibration curve recorded on a stiff non-deformable surface (Fig. 1a). This requires the knowledge of the position of the contact point. In this study, we used eye inspection convoluted with fitting a horizontal line to the baseline; therefore, indentations lower than 100 nm are not considered during the analysis. The obtained force-versus-indentation curves were then fitted to the Hertz model (Lekka et al. 1999; Schillers et al. 2017). In our study, AFM probes with four-sided geometry were approximated by a cone. In such a case, the relation between the load force (*F*) and the indentation depth (*δ*) is

1 $$F\left( \delta \right) = \frac{2}{\pi }\tan \left( \alpha \right) E_{{{\text{eff}}}} \delta^{2} ,$$

where *α* is the open-angle of the cone. For a sphere of radius *R*, the following equation was applied:

2 $$F\left( \delta \right) = \frac{4}{3}\sqrt R E_{{{\text{eff}}}} \delta^{\frac{3}{2}} .$$

*E*_eff_ is the reduced Young’s modulus given by

3 $$\frac{1}{{E_{{{\text{eff}}}} }} = \frac{{1 - \mu_{{{\text{tip}}}}^{2} }}{{E_{{{\text{tip}}}} }} + \frac{{1 - \mu_{{{\text{sample}}}}^{2} }}{{E_{{{\text{sample}}}} }}.$$

When *E*_sample_ << *E*_tip_, the following relationship can be obtained:

4 $$E_{{{\text{eff}}}} = \frac{{E_{{{\text{sample}}}} }}{{1 - \mu_{{{\text{sample}}}}^{2} }},$$

where *µ*_sample_ and *µ*_tip_ are Poisson’s ratio related to the compressibility of the sample and indenting tip.

In our analysis, we set Poisson’s ratio to 0.5 for both polyacrylamide gels and cells assuming that these samples are incompressible. The apparent Young’s modulus was calculated by fitting a Gauss function to moduli distributions (Fig. 1b presents an exemplary histogram obtained for a 5% PA sample measured with OTR4 AFM probe). The center of the distribution denotes the mean value, while the standard deviation is determined from the distribution width. Figure 1c shows the variability of elastic modulus present within the recorded scan area of 6 μ.

### Obtaining the relation between Young’s modulus and indentation depths

In our analysis, we calculated the Young’s modulus as a function of indentation depth varied within a range of 100–700 nm, with a step of 50 nm. Indentation depth below 100 nm was omitted to a possible effect of a misdefined contact point between a tip and a sample surface while larger indentations (above 700 nm might be influenced by the underlying substrate observed as an increase of the modulus values). Starting from the contact point, the Hertz model was fitted to a fragment of the force curve corresponding to a specific indentation (Fig. 1d). The shape of the indenting tip defines the relationship between a load force and indentation depth. The cone (or pyramid) predicts *F* = *B*·*δ*^2,^ while for the sphere (or paraboloid), *F* = *A*·*δ*^3/2^. Both A and B can be re-written in a form *c*·*E*, where *c* is the constant including all the geometrical information together with the Poisson’s ratio and *E* is Young’s modulus (Weber et al. 2019). Frequently, real data recorded during cell indentation rarely fully follow these relations. To elaborate more precisely how the divergence of fitting data with both relations affects the fitting parameters, experimental points (*δ*_*n*_, *F*_*n*_) in the force–indentation curve can be assumed to follow: *F*_*n*_ = *A*·*δ*_*n*_^2−*ε*^ and *δ*_*n*_ = *n*·*δ*_0_, where *δ*_0_ is the indentation difference between adjacent points, *n* is the number of points, *y*_*n*_ is the load force corresponding to the indentation *δ*_*n*_, *A* is the fitting parameter proportional to Young’s modulus, and *ε* is the value expressing how much the cone (or pyramid) approximation differs from the experimental curve. The fitted parameter *B* can be calculated from *F*_*n*_ = *B*·*δ*_*n*_^2^. For *N* experimental points recorded up to chosen maximum indentation:

5 $$B = A \cdot \delta_{0}^{ - \varepsilon } \cdot \frac{{\mathop \sum \nolimits_{n = 0}^{N} \delta n^{4 - \varepsilon } }}{{\mathop \sum \nolimits_{n = 0}^{N} \delta \delta n^{4} }}.$$

Thus, the larger *N* (i.e. fitting data for larger indentation) will generate the lower fitted *B* value (Fig. 1e). Analogously, in the case of spherical (or paraboloidal) assumption of the indenter, the fitted parameter *C* follows the relation:

6 $$C = A \cdot \delta_{0}^{ - \varepsilon } \cdot \frac{{\mathop \sum \nolimits_{n = 0}^{N} \delta n^{3 - \varepsilon } }}{{\mathop \sum \nolimits_{n = 0}^{N} \delta n^{3} }}.$$

The *C* value increases with the data size to be fitted (for larger *N*, the higher fitted *C* is obtained, Fig. 1e). Depending on the theoretical model, the divergence *ε* was calculated. It shows how much the obtained data differs from the assumed theoretical model (cone or paraboloid). In our case, the choosing a cone as an approximation of the AFM tip shape induces its smaller deviations (Fig. 1f).

### Elasticity mapping

Elasticity maps of cells were acquired with the use of JPK AFM equipped with NanoWizard 4 head by employing a classical force volume mode. Rectangular cantilever ORC8 with nominal spring constant *k* = 0.05 N/m, nominal resonance frequency *f*_nom_ = 18 kHz, opening half-angle *α* = 36°, and nominal tip radius of 15 nm were used. We choose ORC8 instead of OTR4 as these cantilevers seems to be more stable when long force volume measurements are conducted. The AFM tip shape of OTR4 and ORC8 is the same. Due to cell heterogeneity, the size of maps varied between 45 µm × 45 µm and 50 μm × 50 μm, but, always, a size of a single-pixel kept being 1 µm^2^. Elasticity maps were recorded with the approach/retract speed of 8 μm/s and load force 10 nN. The time needed to record a single map varied from 45 to 70 min.

### Scanning electron microscopy

The SEM images of the used cantilevers were recorded using the FEI Quanta FEG-SEM in low vacuum conditions. The electron beam was operating at 5 kV accelerating voltage, 5 nA current, and a working distance of about 5 mm. Images of AFM tips were captured using the secondary electrons signal for only one set of cantilevers (one image per one cantilever).

### Statistical analysis

All calculations and statistical analyses were performed using OriginPro 2015. Data are represented as the mean ± standard error obtained from all measurements. Statistical significance was evaluated using two-sample Student’s *t* test for testing the equality of the means between two populations, assuming various numbers of samples analyzed (using OriginPro 2015). All statistical tests were two-sided and *p* values of < 0.05 were considered statistically significant.

## Results and discussion

### Geometrical and mechanical properties of AFM probes

**Fig. 2:**
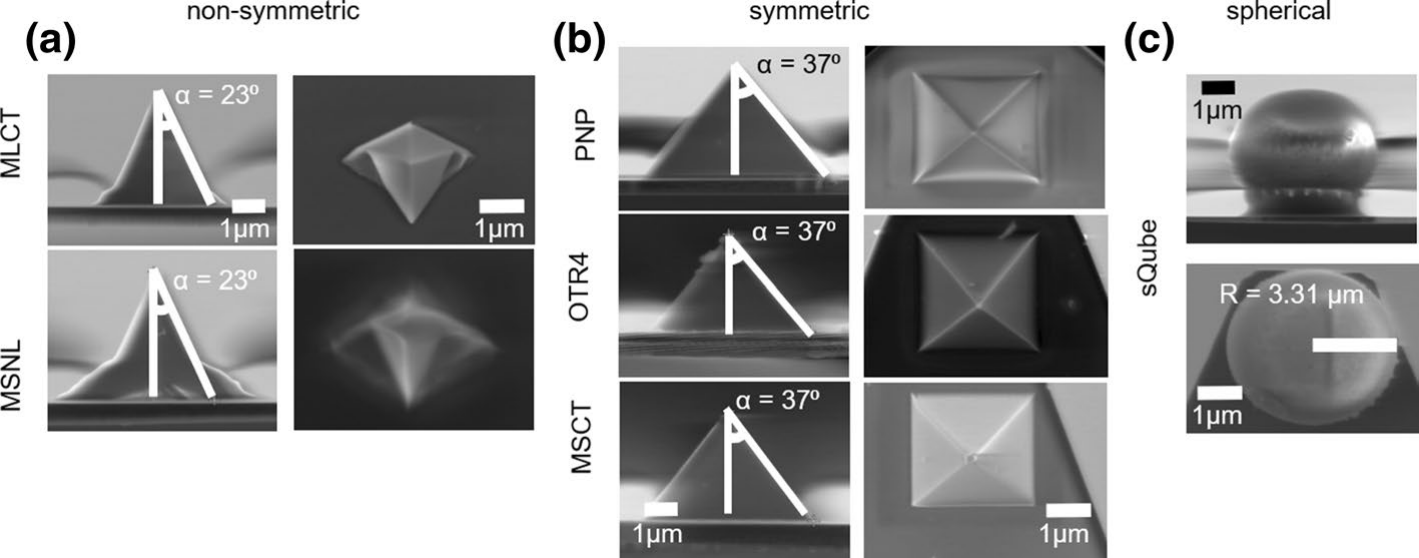
Representative SEM images of non-symmetric (**a**), symmetric (**b**), and spherical (**c**) AFM probes used in measurements of mechanical properties of hydrogels and cells

AFM probes chosen for our study were non-symmetric (MLCT, MSNL) and symmetric (PNP, MSCT, OTR4) pyramidal probes. As a reference, a spherical probe (sQube) was used. The geometry of probes (Fig. 2) was visualized using scanning electron microscopy (SEM). Based on SEM images, the main parameters describing probe geometry, i.e., height, opening angle, and sphere radius, were determined with 1% accuracy. The height of symmetric pyramids was about 3 µm while non-symmetric ones were characterized by a height of about 4 μm. The radius of spherical probes used was found to be 3.31 ± 0.03 µm corresponding to a height of about 6.4 µm (Table 1). Spring constants of the cantilevers were calibrated using the thermal noise method in the air. For each cantilever, the resonant frequency was recorded with an accuracy below 2.5%, and then, it was used to calculate spring constant using a protocol described elsewhere (Schillers et al. 2017). All pyramidal cantilevers reveal larger cantilever spring constants as compared to nominal values, while for the spherical probe, the cantilever spring constant was smaller. Such a relation is fully accidental as spring constants depend strongly on production batch.

### Mechanical properties of polyacrylamide gels

**Fig. 3:**
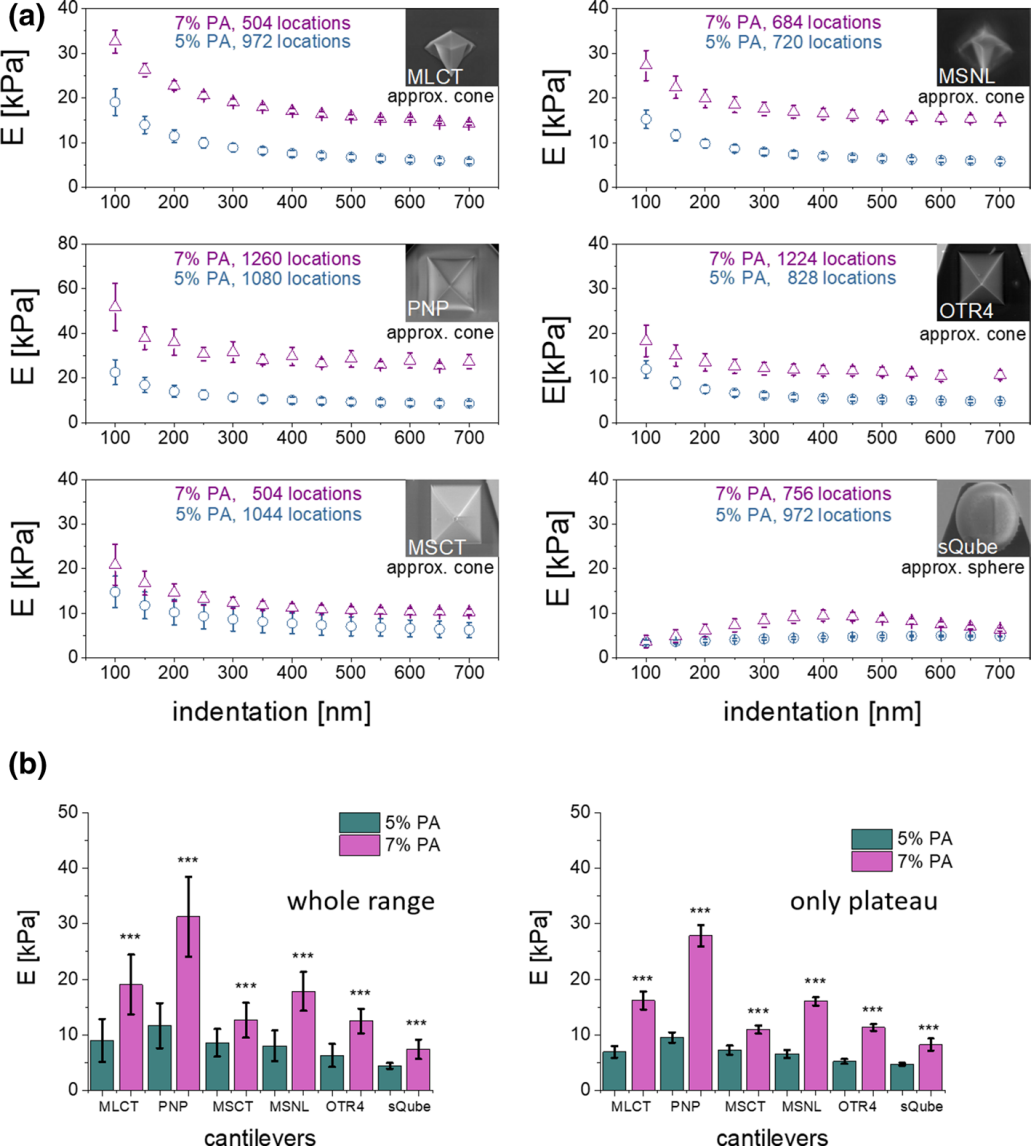
**a** Relations between Young’s (elastic) modulus and the indentation depth, obtained for PA hydrogel samples measured with a set of AFM probes. Each point denotes a mean modulus ± standard error obtained from all measurements (number of locations is pointed in each plot). **b** Comparison of Young’s modulus means calculated for a whole and plateau region of the indentation depth (error bars represent the standard error of the mean, asterisk (***) denotes *p* value < 0.001 quantifying the statistical difference between the corresponding pair of 5% and 7% hydrogels. Note *Y* scale for PNP is twofold larger than for the other cantilevers)

Knowing that cells are highly heterogeneous in their internal structure, in the first steps, polyacrylamide (PA) gels were applied, as they constitute samples of high homogeneity. To mimic the elasticity of bladder cancer cells (Lekka et al. 1999, 2019; Lin and Horkay 2008; Ramos et al. 2014), two groups of gel samples, containing 5% and 7% of acrylamide monomers, were probed with all types of cantilevers. All samples were measured when immersed in deionized water after 24 h post-polymerization (to minimize swelling effect). The Young’s modulus was calculated using Hertz–Sneddon mechanics and plotted as a function of indentation depth. Results show that for all pyramidal cantilevers, Young’s modulus decreases with increasing indentation depths, reaching a plateau for indentations above 300 nm (Fig. 3a). We could identify two effects responsible for the shape of these relations, namely, contact point determination and the way of fitting the model to the data (Fig. 1d). The former reason is mathematically obvious—a contact point position located within the region with higher deflections will deliver a larger Young’s modulus. The latter stems from the fact that the divergence of fitting data for the conical approximation of the AFM tip shape is larger for small indentations (Fig. 1e, f). The relation between Young’s modulus and indentation obtained from data indented with a spherical probe was almost flat. To evaluate how large is the effect in the overall mechanical properties of polyacrylamide samples, Young’s modulus was calculated for a whole and plateau regions of the indentation depth (Fig. 3). Results reveal that regardless of the tip geometry, for such highly elastic material like PA hydrogels, mutual relations between mechanical properties of the studied hydrogel samples were preserved, also regardless of the indentation depth region taken for the analysis. The moduli range for soft samples (5% PA) varied between 4 and 12 kPa while for stiffer samples (7% PA)—from 7 to 31 kPa. The lowest values were obtained from indenting experiments with spherical probes (4.43 ± 0.52 for 5% PA and 7.41 ± 1.77 kPa) in agreement with already published data (Rico et al. 2005; Carl and Schillers 2008; Managuli and Roy 2017; Alcaraz et al. 2017; Sokolov and Dokukin 2017; Jorba et al. 2017; Giménez et al. 2017). Although all moduli values originating from indentation experiments carried out using pyramidal cantilevers were larger, they were not uniform. As this stems from the shape of the indenting pyramid, thus, cantilevers were separated into non-symmetric and symmetric probes. Non-symmetric probes (MLCT and MSNL) deliver similar modulus values of 8–9 kPa for 5% PA and 17–19 kPa for 7% PA. This could be an effect of optimization of the shape of these probes for indentation, as they are supposed to indent the sample perpendicularly. Moduli variations were much larger for symmetric probes, for which values of 6–12 kPa and 12–32 kPa were obtained for 5% and 7% PA, respectively. MSCT probes bring the corresponding Young’s moduli close to results obtained from non-symmetric cantilevers, i.e., 8.56 ± 2.47 kPa but only for softer gel samples. Two other cantilever types (PNP and OTR4) deliver values that deviate from that of MSCT, however, there is no pattern observed for these changes. Mechanical properties of soft PA gel samples (5% PA) were described by three different moduli values, i.e., 8.56 ± 2.47 kPa (MSCT), 6.29 ± 2.08 kPa (OTR4) and 11.63 ± 4.05 kPa (PNP). Stiffer gels (7% PA) were characterized by another set of values 12.64 ± 3.15 kPa (MSCT), 12.51 ± 2.22 kPa (OTR4) and 31.29 ± 7.18 kPa (PNP). This is a bit surprising, as all symmetric cantilevers have similar geometrical parameters: opening angle, height, and radius of curvature. Excluding the effect of the fit and contact point determination by considering only the plateau region did not unify the results. The moduli variability between data obtained for MSCT, OTR4, and PNP cantilevers remained. The only explanation considers the hydrogel’s response to a load rate, which changes with time during indentation and the applied load. This is dependent on the contact area with the probing tip. Altogether, these results demonstrate the shape of the AFM may play a role, even if mechanically homogenous samples are measured and support that it is important to use one type of cantilever during a set of measurements.

### Mechanical properties of bladder cancer cells

**Fig. 4:**
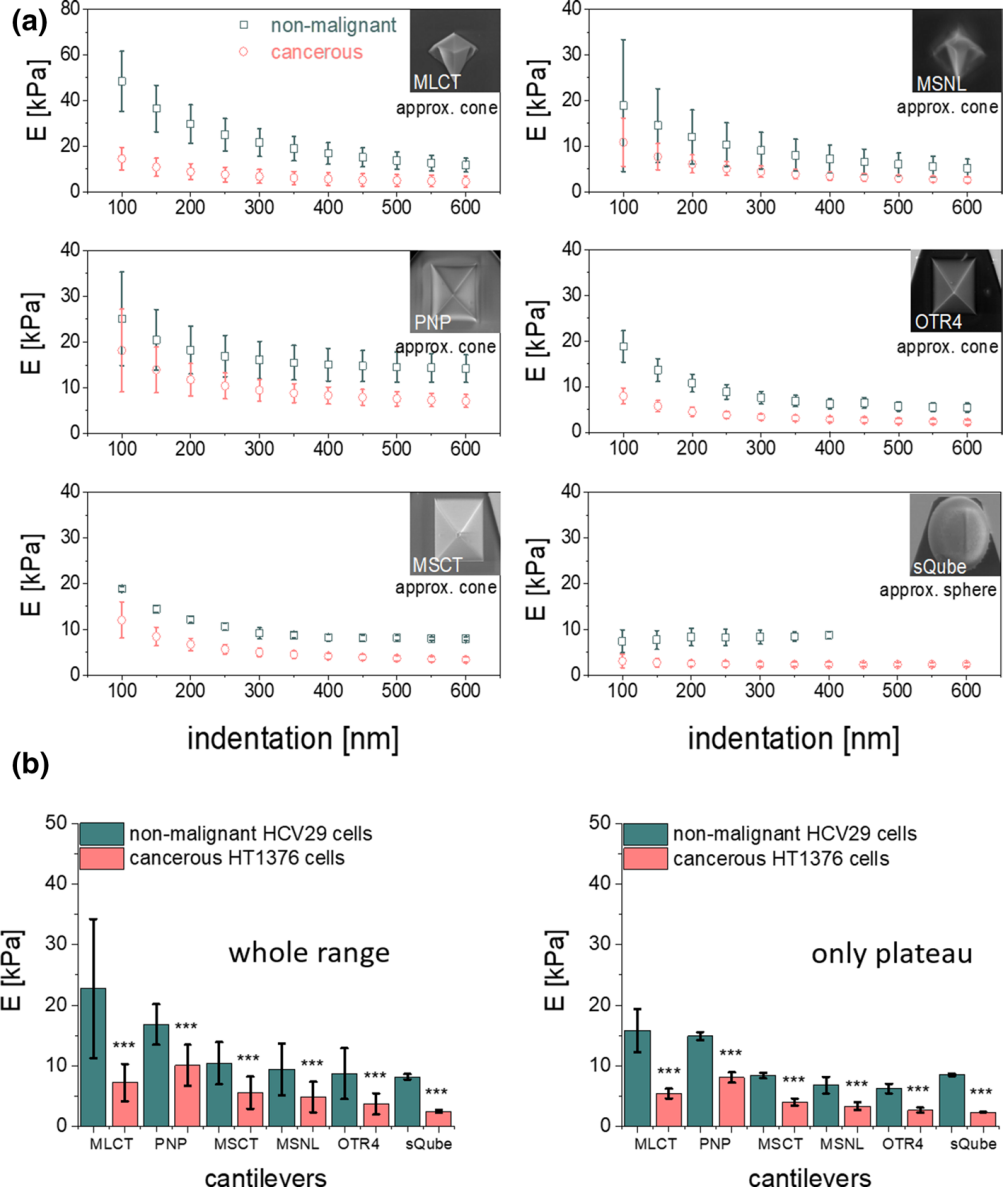
**a** Relations between Young’s (elastic) modulus and the indentation depth, obtained for bladder cancer cells, measured with a set of AFM probes. Each point denotes a mean modulus ± standard error obtained from all measurements. **b** Comparison of Young’s modulus means calculated for a whole and plateau region of the indentation depth (error bars represent the standard error of the mean, asterisk (***) denotes *p* value < 0.001 quantifying the statistical difference between the corresponding pair of reference and cancerous cell line. Note *Y* scale for MLCT is twofold larger than for the other cantilevers)

To elaborate on the effect of the shape of the probing tip in cell-related measurements of mechanical properties, human bladder cancer cells were next studied. In comparison with PA hydrogels, cells are highly mechanically heterogeneous, as they are structurally heterogeneous. Analogously as for PA gels, measurements were conducted for three groups of cantilevers on non-malignant cell cancer of ureter (HCV29) and bladder carcinoma (HT1376) cells. The obtained results show that, similarly as for hydrogel samples, Young’s modulus varies as a function of the indentation depth (Fig. 4a). Here, in addition to the approximation of the AFM tip shape and contact point determination, the high degree of mechanical heterogeneity of the superficial layers of the cell can contribute to the shape of the relation. Importantly, this does not affect the deformability difference between reference (non-malignant HCV29) and cancerous (HT1376) cells. Independently of the cantilever type chosen for the study and indentation range taken for the analysis, always cancer cells are softer. Results recorded for data collected with the spherical probes were smaller (8.19 ± 0.46 kPa and 2.47 ± 0.25 kPa for HCV29 and HT1376 cells, respectively) as compared to moduli calculated from data recorded with pyramidal cantilevers showing the corresponding values above 8.5 kPa and 3.5 kPa. Focusing on symmetric and non-symmetric cantilevers, there is no correlation with probe shape nor with cantilever spring constant. This probably stems from the structure, heterogeneity, and viscoelastic nature of the cells. In our next step, we ask ourselves whether the choice of a specific geometry of the AFM probe and indentation range affects the difference between reference and cancerous cells. In the case of considering a whole indentation range (Fig. 4b), cancer cells were of 68%, 48%, 40%, 46%, 57% and 67% more deformable than HCV29 cells when measured with MLCT, MSNL, PNP, MSCT, OTR4 and sQube types of cantilevers. In the case of considering indentations above 300 nm, the Young’s moduli of cancer cells were of 66%, 50%, 46%, 52%, 57% and 72% smaller than that for HCV29 cells when measured with MLCT, MSNL, PNP, MSCT, OTR4 and sQube types of cantilevers. These results show that by choosing a specific indentation range, it is possible to enhance the difference between reference and cancerous cells from 2 to 6%. This could have a significance when a smaller difference in cell deformability occurs.

Our results allow us to conclude that for diagnostic purposes, all the types of AFM probes are suitable. This partially agrees with the observations by Kim et al. (2013) who studied mechanical properties of normal hepatocytes (THLE-2) and hepatocellular carcinoma cells (HepG2) with a conical tip end and a bead probe. They state that the conical shape of the AFM tip delivers higher elastic modulus values and they also claim that conical probes are more suitable for comparative studies. However, Kulkarni et al. (2019) have recently shown that in certain biological conditions (human pancreatic cancer cell line, Panc1), one might observe the inverse relationship. It must be noted that indenting cells with sharp probes results in higher Young’s moduli than for cells indented with a spherical probe. This has already been reported by Carl and Schillers (2008), who compared indentation data of living cells collected with sharp and various spherical probes. The results show that the size of the colloidal probe does not influence the elasticity results and that indenting the cells with sharp tips leads to higher Young’s moduli regardless of the cell type. In addition, the standard deviation of modulus estimated for sharp probes is larger as compared to the spherical probe. This is related to the small radius of curvature of pyramidal probes enabling them to resolve a local (nanometer scale) spatial resolution. Consequently, such AFM measurements reveal cell-related variability resulting from the structural components of the cell. The passive mechanical response of cells is determined mainly by the cell membrane, the cytosol, and the cytoskeleton. The cytoskeleton is a mesh-like structure of actin filaments, microtubules, and intermediate filaments which causes anisotropic mechanical response due to the presence of stress fibers and attachments to the membrane.

### Elasticity maps recorded with a pyramidal AFM probe

**Fig. 5:**
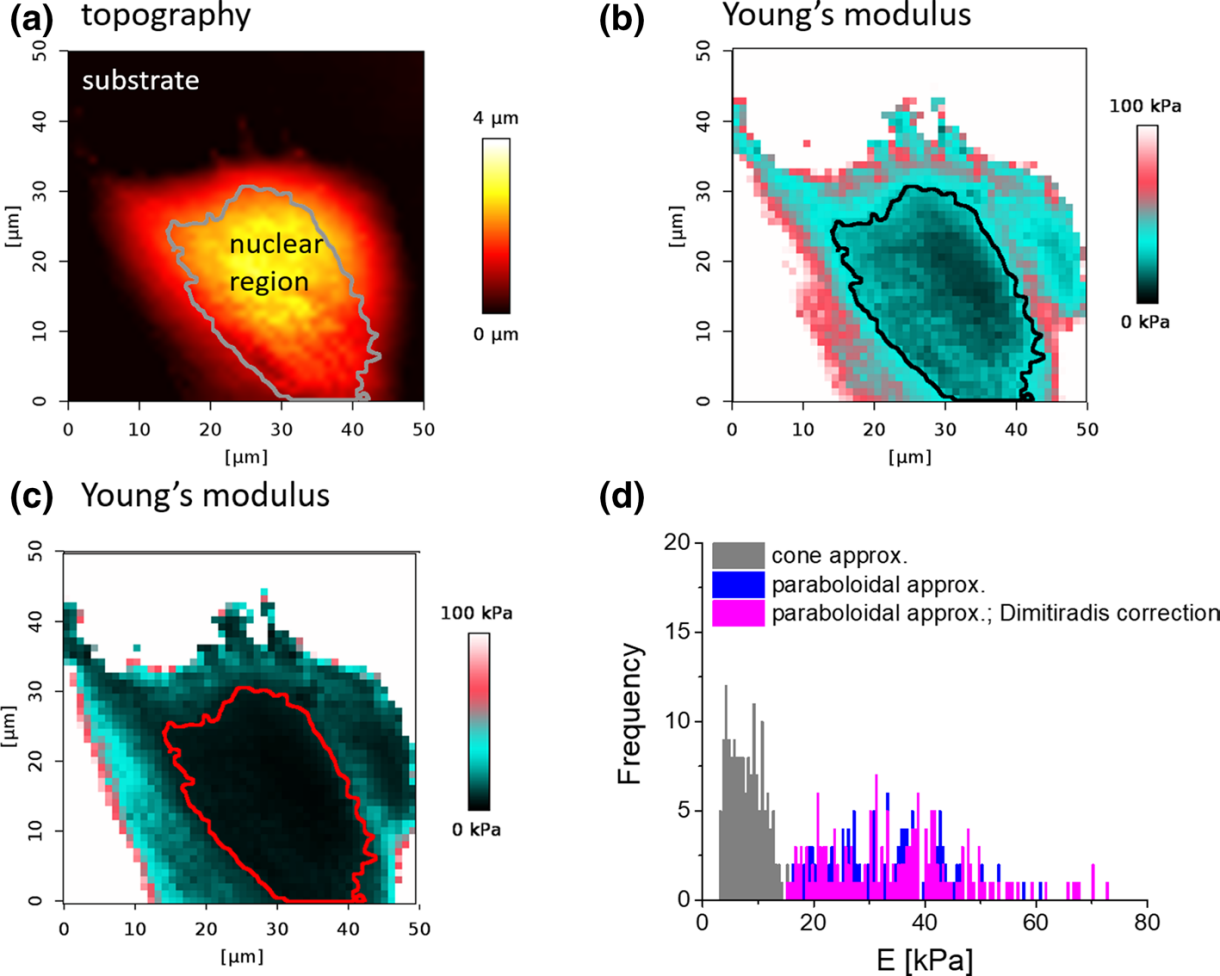
**a** Topography of a single HCV29 cell acquired using force volume like approach. **b**, **c** Recorded force curves were fitted either by a paraboloidal or a conical approximation of the AFM tip. **d** Young’s modulus histograms obtained for data recorded within the nuclear region of the cell using both cone and paraboloidal approximation of the AFM tip

To analyze the effect of the choice of theoretical models, in our next step, we compare the outcome of the Young’s modulus determination for data recorded with a pyramidal probe, that shape was approximated either by a cone or a paraboloid. Acquired force curves were analyzed using two approximations of the shape of the AFM tip: cone and paraboloid (Fig. 5a, c). Young’s modulus calculated for paraboloidal approximation of the tip shape is larger as compared to data obtained using a cone (Fig. 5d). This is related to the mathematical function applied, i.e., *F* = *A*·*δ*^1.5^ instead of *F* = *A*·*δ*^2^. Instead of conical approximation, the data could also be approximated by a four-sided pyramid. Since the equations relating a load force and indentation follows the function *F* = *A*·*δ*^2^, the main difference between a cone and a pyramid is a constant factor. In such a case, the divergence between the raw data and the fitted curve will remain constant. A change will be observed in the fitted factor *A*.

Analyzed data showed similarities in the calculated elasticity maps. In both cases, modulus changes depending on the force curve location. Single cells show a softer region around cell nuclei and are stiffer at the cell periphery. Higher Young’s modulus may also be a result of the presence of a stiff substrate used for cell culture. In such a case, the largest values are observed at the peripheral part of the cell, where their height is smaller than that in the nuclear regions (Chiou et al. 2013). The analysis focused on the force curves recorded only within a nuclear region shows a large variation in Young’s modulus in the case of paraboloidal approximation of the AFM tip (Fig. 5d). To evaluate whether such observation results from the effect of substrate, we applied finite thickness sample correction proposed by Dimitriadis et al. (2002). Initial assumption that this stem from the substrate effect fails. Both histograms showing Young’s modulus distribution before and after finite thickness correction overlap (Fig. 5d). Therefore, we assumed that the observed large modulus variability originated from the mismatch between recorded data and chosen mechanical model which was confirmed by looking at the R-squared (a statistical measure describing how well a model was fit to the data). Its values change from 0.2 to 1 for the fits assuming paraboloidal approximation of the tip shape and from 0.7 to 1 for fitting with an assumed conical approximation of the tip shape.

## Conclusions

By measuring the mechanical properties of hydrogels and cells, we elaborated on the effect of indenter shape on determined values of Young’s modulus for soft samples like polyacrylamide gels and living cells. In our study, the elastic modulus was determined from measurements with a wide range of AFM probes, and plotted as a function of the indentation depth. For highly elastic soft samples such as polyacrylamide gels, its value was not constant as expected; oppositely, it reveals an exponential-like decrease showing a plateau for large indentations, which stems from the way the data are analysed but also it may include the load rate effect. Averaging over a whole sample or only within the plateau range showed a significant difference in elastic properties of softer and stiffer hydrogels. The magnitude of changes was dependent on the cantilever used, more precisely on the shape of the probing tip. Spherical probes deliver the smallest Young’s modulus value (according to already reported data), while pyramidal probes (both non-symmetric and symmetric) reveal a probe-dependent relation. Switching to results obtained from heterogeneous samples like cells, our analysis demonstrated that non-malignant HCV29 cells are stiffer than the cancerous HT1376 cells independently of the AFM cantilever type used. Analogously as for hydrogel samples, the smallest modulus was obtained for spherical probes, while for pyramidal probes, large moduli variability was observed. However, its character is different as compared to hydrogel samples and is linked with the structure of the cells or, as it was demonstrated for elasticity maps, with the fact that cells due to their viscoelastic nature follow neither with *F* = *A*·*δ*^1.5^ nor with *F* = *A*·*δ*^2^ functions. Altogether, these results show that the importance of the choice of cantilever type for studies of the mechanical properties of cells. In particular, the tip shape of the AFM probes affects the determination of Young’s moduli, even if mechanically homogenous samples are measured and support that it is important to use one type of cantilever during a set of measurements.

## Data Availability

Data are available upon reasonable request.
